# Forgiveness and Identification

**DOI:** 10.1007/s11406-016-9688-9

**Published:** 2016-03-22

**Authors:** Geoffrey Scarre

**Affiliations:** Department of Philosophy, Durham University, 50 Old Elvet, Durham, DH1 3HN UK

**Keywords:** Forgiveness, Proxy forgiveness, Identification, Wrongdoing, Definite description

## Abstract

Philosophical discussion of forgiveness has mainly focused on cases in which victims and offenders are known to each other. But it commonly happens that a victim brings an offender under a definite description (e.g. ‘the boy who kicked his football through my window’) but does not know to which individual this applies. I explore some of the conceptual and moral issues raised by the phenomenon of forgiveness in circumstances in which identification is incomplete, tentative or even mistaken. Among the conclusions reached are that correct and precise identification of the offending individual is not essential for forgiveness to take place; that an offender can, under certain strict conditions, be said to be forgiven *by proxy* where the victim has misidentified the offender and ‘forgiven’ the wrong person; and that proxy forgiveness of this sort is not subject to the objections commonly levelled against ‘proxy’ or ‘third-party forgiveness.’

Much of the philosophical discussion of forgiveness has focused, for understandable reasons, on cases where a victim offers or refuses forgiveness to an offender whose identity she knows. These are the cases in which the potential of forgiveness to restore damaged relationships, allay hurt feelings and assist new starts is at its greatest, and the cognitive, emotive and moral aspects of forgiveness that enable it to do these things have received due attention in the ever-growing literature. Yet it is common experience that much wrongdoing takes place behind a veil of anonymity, with many offenders wishing to keep their identity unknown from the world in general or from their victim in particular. Such wrongdoers may be moved by shame or embarrassment but more commonly are seeking to avoid the customary penalties for bad behaviour. By acting in the dark, they compound their offence, leaving the victims with a sense of frustration at not knowing whom to blame. Who was it, you wonder angrily, who scraped your car in the parking lot, or sent that slanderous tale about you to the local press, or maliciously reported you to your boss for being ten minutes late to work? What, if any, form can or should forgiveness take when the victim does not know who the wrongdoer is? In this paper I explore some of the conceptual and moral issues for forgiveness that arise in circumstances in which identification of the offender is incomplete, tentative or even incorrect: a topic of some importance that has not so far received much philosophical attention.

Note, to begin with, that there are different senses of the expression ‘does not know who the wrongdoer is.’ One may not know an offender’s name, or any other personal information about him, but be able to single him out under a definite description, e.g. ‘the man in the red hat who threw the stone at me.’ If you consider that the stone-throwing took place during a street protest by people who had a genuine grievance to air (even if it did not justify violent methods), and that the man may not have been particularly aiming at you but throwing stones at random, you may feel you can forgive him, though he remains for you a nameless individual. More interesting are cases in which one similarly brings the offender under an identifying definite description, but where one cannot (as one can with the stone-thrower) pick out which specific person the description fits. Even this level of ignorance need not preclude forgiveness. Famously it did not in the case of Gordon Wilson, the father who magnanimously granted forgiveness to the unknown member of the IRA who planted the 1987 terrorist bomb in Enniskillen which killed his daughter. And indeed it is always possible to bring a potential target for forgiveness under the most minimal description: ‘the person who committed offence O.’ Yet where that is all that the victim knows or can infer about the wrongdoer, then forgiveness is severely hampered by having nothing to work on. Gordon Wilson, in forgiving the unidentified IRA bomber, was able at least to infer the probable motives of the bomber from his knowledge of the embittered politics of Northern Ireland.

So a victim of wrongdoing might not know the name of her offender or to which individual the definite description under which she identifies him applies, yet still know enough *about* him to be able to consider forgiveness. In some cases a third party may open a line of communication between offender and victim, without revealing the former’s identity to the latter. Or the third party could inform the victim that her offender is sincerely repentant and desiring forgiveness. The victim may not feel able wholly to extinguish her resentment so long as the wrongdoer preserves his anonymity; this reluctance may put his good faith in doubt and impede reconciliation. Nevertheless, it would certainly be too strong to insist that forgiveness is either impossible or improper unless the victim of wrongdoing knows just which individual has offended. This is so even in some serious cases (though perhaps not in all – a point I shall return to at the end). If one learns from a reliable source that the person whose dangerous driving knocked down one’s child is deeply remorseful, one may feel able to forgive him even without knowing his identity; and no one is entitled to say one would be wrong. 1.

Incidentally, definite descriptions under which targets of forgiveness are identified are referentially transparent. Suppose that you have brought yourself to forgive the otherwise unknown individual who stole your lunch-box while you were on the way to work this morning. The person who stole the lunch box was in fact the man who sat beside you on the 8.05 train. You have therefore forgiven the man who sat beside you on the train, although you do not realise it. But suppose further that the man who stole the lunch box was also the man who rudely pushed past you on leaving the station. Have you therefore forgiven the man who rudely pushed past you at the station exit? Yes, you have – but for stealing your lunch box, not for pushing past you; that separate offence calls for separate consideration. If you should decide to forgive him for his rudeness (perhaps, you reflect, he was late for an important meeting), you are also forgiving for *that* offence the man who stole your lunch-box.

That forgiveness does not require (though it will usually be facilitated by) the victim’s knowing just which individual is the author of the wrong she has suffered, leaves open that a victim may occasionally misidentify the individual she has forgiven. Consider this case. Some boys are playing football in the street outside your house and one unintentionally kicks the ball through your study window, shattering the glass. When you look out you see the boys running away and you cannot tell which of them performed the fatal kick. At first you plot dire retribution, but gradually your anger cools. You consider that the breaking of the window was merely an unlucky accident and that the local boys have to play in the street because there is no nearby field or playground for them; also that they are, on the whole, a well-behaved, polite set of youngsters. Eventually your ill-feelings evaporate, and you forgive the boy (whoever he was) who broke your window. Afterwards a neighbour who witnessed the incident tells you that it was Craig Potter who kicked the ball. You therefore conclude that it is Craig Potter whom you have forgiven. But the information is incorrect (the neighbour is short-sighted), and it was really Jerry Lindley who broke the window. So which of the two boys have you actually forgiven? Jerry Lindley, surely, although you do not know it. The reason is that your forgiveness is targeted via the description ‘the boy who broke my window,’ and that description applies to Jerry and not to Craig.

Now imagine a different scene. Upon hearing the breaking of glass, you run to the window and are in time to see the guilty youth standing appalled at his deed, before all the boys run off down the street. The malefactor you identify as Craig Potter. But this time it is *you* who are short-sighted, and the boy you saw was actually Jerry Lindley. As before, you are initially angry but gradually your mood softens. Finally you forgive Craig Potter and resolve to tell him so when you meet him. Here you do not identify the object of your forgiveness via the definite description, ‘the boy who broke my window,’ but bestow your forgiveness directly on Craig Potter. Therefore it cannot this time be said that you are really forgiving Jerry Lindley on the strength of his being the actual referent of ‘the boy who broke the window.’

But there is another interesting possibility here. Suppose that you would have been equally disposed to forgive Jerry as Craig, if you had believed the former to have been the culprit. And were you subsequently to learn from another witness with better eyesight than your own that Jerry, not Craig, broke the window, you would not feel that the process of forgiving had to be gone through again. Indeed, you might feel that, to all intents and purposes, you had *already* forgiven Jerry, when you mistakenly forgave Craig. In effect, by forgiving Craig, you had already forgiven Jerry *by proxy*.

For this last claim to be plausible, three conditions need to be satisfied:

- that Jerry has done the deed you are forgiving Craig for;
- that were you to learn that Jerry, not Craig, was the actual offender, you would forgive him without more ado;
- that the reason you would forgive Jerry without more ado is that the precise identity of the offender (Craig or Jerry) is not salient to your readiness to forgive or refuse forgiveness for the offence in question.

Condition c) is required in order to rule out this kind of case: although you know Craig Potter to be a well-behaved youth who generally respects other people’s property, you regard Jerry Lindley as a cheeky young devil whose offences are legion; hence you would be much less ready to forgive Jerry than Craig. In this scenario, it could not be said that, in forgiving Craig, you had forgiven Jerry by proxy. But where you would be equally ready to extend forgiveness for breaking your window to either Craig or Jerry, the claim that you actually forgive Jerry *by* forgiving Craig is much more intuitive. Even if you are wrong in believing Jerry to be a more depraved youth than Craig, the fact that you *believe* it is enough to prevent your forgiving Jerry by proxy. Regrettably, it would also block proxy forgiveness if your preparedness to forgive Craig but not Jerry was based on unreasonable grounds, such as racial or religious prejudice.

Suppose that, where conditions a) to c) held, you were to tell Craig Potter that you forgave him for breaking your window and that Craig, wishing to shield his friend, accepted both the blame and the forgiveness. Afterwards he tells Jerry what you have said to him, and of your forgiving attitude. Jerry may be unhappy that his friend has taken the blame and come to you to make his own apology. Still, Craig’s words may convince him that his offence has already been forgiven. For the grounds on which you forgave Craig apply to him equally, and your misidentification of the offender was not salient to your forgivingness.

Would it not be better if your forgiveness had taken a more straightforward course, and Jerry been rightly identified as the offender? Yes, in so far as it is intrinsically undesirable to attribute moral blame to the wrong person, even if no bad practical consequences issue from the mistake. But the question is whether, in this less than perfect scenario, Jerry is nevertheless forgiven by proxy. Perhaps there is no simple answer to this question. As Charles Griswold has pointed out, the concept of forgiveness lacks sharp boundaries, and there are marginal and uncertain cases of forgiveness as well as paradigmatic and common ones (Griswold 2007: 103). Some may wish to rule out a priori the idea that one person can ever be the actual recipient of forgiveness mistakenly bestowed on another. Against this conceptual hard line, it may be noted that in a situation like that of Jerry and Craig, neither victim nor offender may feel that anything essential remains to be done in the way of forgiveness after the initial granting of forgiveness (albeit to the wrong person) has occurred. While it would be good if the mistaken identification were later corrected, this does not seem to be necessary to the true offender being forgiven.

This kind of ‘proxy’ forgiveness is not to be confused with the sort which has been much more often discussed in the literature, that in which one person purports to forgive on behalf of another. On the whole, philosophers have been sceptical about the possibility of ‘third-party’ or ‘proxy’ forgiveness, maintaining for a variety of logical and ethical reasons that only the victims of wrongdoing have the requisite status to forgive those who have injured them. Logically, if forgiveness involves relinquishing the first-personal resentment that the victim feels on being the target of offending (the sense of grievance that one is oneself on the receiving-end of another’s wrongful action), then only the victim can relinquish *that* resentment (though others who are resentful on one’s behalf will have their own resentment to deal with). And many philosophers have rejected third-party forgiveness on the ground that only victims have the moral standing to forgive, any other persons’ attempts to do so being an usurpation of the victim’s prerogative 2.

Proxy or third-party forgiveness appears objectionable because it sidelines the original victim, the course of ‘forgiveness’ from giver to recipient passing her by. Proxy forgiveness that concerns instead the *recipients* of forgiveness involves no such sidelining. Where Jerry receives your forgiveness via the forgiveness you grant to Craig, your forgiveness passes through Craig* en route* to Jerry. In this way, forgiveness runs its full and proper course from victim to offender, in spite of the fact that you, the forgiving party, misidentify the person to be forgiven. Whereas in the act (or process) of forgiving, anyone other than the victim is *the wrong person* to grant forgiveness, in the case where forgiveness is granted via a proxy the *right persons* – victim and offender – are brought into the relationship of forgiver and forgiven. Here the proxy is not an end-term of the forgiveness relationship (as she would be if forgiveness by third parties were possible) but – more properly – a middle term or intermediary.

What does, however, seem to be impossible is to forgive X by forgiving Y when you *know* X to be a proxy. It is possible to forgive Jerry via forgiving Craig so long as you believe the errant footballer to have been Craig; but once you learn the real facts, then your forgiveness is bestowed directly on Jerry. Forgiveness never knowingly travels through a proxy. (Of course, one may employ an intermediary to convey a *message* of forgiveness to a wrongdoer, and it may occasionally be appropriate to express one’s forgiveness by showing favour to people who are near or dear to the offender. A third party may also be an advocate for an offender, or help one by persuasion or other methods to achieve a state of forgiveness. But in none of these situations does one forgive the wrongdoer via forgiving someone else.) 3 Nor is one forgiving via a proxy in the case where one is unsure whether one has identified the culprit correctly and expresses one’s forgiveness in the form, ‘I forgive X for Φ-ing – and if it wasn’t X, then whichever of his associates it was.’ If X is not the offender, neither is he a proxy for the offender; rather, one’s forgiveness has a *disjunctive* target: one forgives X *or* someone else, the real offender.

Another situation in which proxy forgiveness may be taken to occur is this. X and Y together offend you by Φ-ing, but you are unaware of Y’s participation and think that X acted alone. Later, on witnessing X’s evident repentance, you forgive him. But suppose that Y, his co-offender, is equally repentant and that, had you known of his participation and repentance, you would have forgiven him just as you have forgiven X. It is reasonable to think that the forgiveness you grant to X extends to Y as well, and that by forgiving X you have forgiven Y too. In this case, X is both a target of forgiveness in his own person and a proxy for Y. It might be objected to this analysis that if you have no idea of Y’s participation in the offence (or possibly even of Y’s existence), to speak of your *forgiving* him, even via a proxy, sounds fanciful. This objection has most force if your main reasons for forgiving X are highly specific in their relation to X and have little application to Y: for instance, you are impressed by X’s plainly genuine and deep contrition, by the heartfelt apology he makes you, and by his favourable reputation with other people. The new (or renewed) personal relationship you strike up with X is advanced and consolidated by your forgiveness of him for Φ-ing, but since there has been no similar development in regard to Y, it is not very plausible to say that he is forgiven via X’s forgiveness. But the objection has rather less force in a different case. Imagine that your chief grounds for forgiving X are supplied by certain descriptions that you take to apply to him: for instance, he is the sort of person who acts first and thinks afterwards, a youth at a difficult age, a member of an impoverished community with many social problems, etc. If these descriptions, or a substantial number of them, apply also to Y, then it becomes more credible to claim that when you forgive X, you simultaneously forgive Y by proxy.

Yet will there not always be something imperfect and unsatisfying both about proxy forgiveness and about non-proxy forgiveness where the object of forgiveness is identified only by one or more definite descriptions that apply to an individual whom one cannot specify (or can at best pick out only as an anonymous body in a crowd, as in the case of the stone-throwing man in the red hat)? In both situations, the person granting forgiveness is ignorant of the individual identity of the person (or persons) to whom she is granting forgiveness. This ignorance places obvious limitations on the possibilities for advancing reconciliation and mutual trust between the parties, not to mention friendship or any closer inter-personal relationships. This is forgiveness-at-a-distance which does nothing to reduce distance.

However, the extent to which the lack of potential of forgiveness of this sort to foster inter-personal relationships renders it ‘unsatisfactory’ should not be overstated. Often there will be no desire on the part of the victim to form or resume any close relationship with the offender; and while, following particularly serious offences, it may help the victim to attain a sense of closure if she can forgive the offender, such closure may be incompatible with any future contact. It is therefore too strong to hold that forgiveness is always imperfect or incomplete if it fails to facilitate the dawning of a period of warm relations between victim and offender; sometimes, forgiveness that brings closure is all that is required. Nevertheless, it may be doubted whether forgiveness of some more significant offences, especially offences against the person, is possible so long as the identity of the offender remains unknown. It is hard, for example, to imagine a victim of rape feeling remotely able to forgive her rapist if she does not know precisely who he is, no matter how many definite descriptions she can bring him under. It is also more difficult to suppose that a rapist can be forgiven by proxy, in a manner analogous to that in which in our example Jerry was forgiven via the forgiveness mistakenly bestowed on Craig; in view of the intimate nature of the offence, our intuitions are more likely to demand that identification of the offender should be correct. The same thing is true of many other serious offences that constitute personal affronts (as distinct from more ‘impersonal’ crimes such as the theft of valuables or vehicles, or internet fraud).

Finally, it should be noted that even where the veil of offender-anonymity is not lifted, this need not preclude all moves to establish a warmer or better relationship between victim and offender. An offender who suffers acute embarrassment about revealing his identity to his victim yet is withal genuinely repentant may, both before and after being granted forgiveness, convey his apologies through a third party, make reparations, send gifts and good wishes, and do all he can to establish mutually beneficial relations short of revealing his identity. This would admittedly be an unusual situation, and one that falls short of the highest ideal of mutual transparency; but it helps to point up the multiform nature of the moral phenomenon we term ‘forgiveness’, and the endless variety of its dynamics.^1^

Notes

1. What if one does not know whether the dangerous driver is remorseful or not? This depends on the view one takes on the question of whether forgiveness is improper unless the victim knows or has good reason to believe that the offender is sorry for his offence. For a defence of unconditional forgiveness, see Garrard and McNaughton 2003, 2010. Gordon Wilson’s unconditional forgiveness of his daughter’s killers has been regarded as saintly by many, yet a contrary view holds that (justifiable) resentment is relinquished too soon while the offender remains unrepentant. I shall not pursue this debate further here, though it is worth noting that forgiveness is unlikely to bear much fruit in the form of bridge-building and reconciliation where an offender is unrepentant.
2. For a cogent statement of the arguments against forgiveness by third parties see, amongst much other literature, Govier and Verwoed 2002; Holmgren 1993, 2012; Walker 2013. For opposing views, see. e.g., Neblett 1974; Pettigrove 2009, Pettigrove 2012.
3. The claim that one cannot forgive X via forgiving Y when one knows Y to be a proxy may, however, seem open to challenge in the following scenario (for which I am indebted to an anonymous reviewer for the journal). A victim who wants to forgive a serious offender but is having trouble doing so might engage in a process of counselling in which the counsellor play-acts the role of the offender until, eventually, the victim finds herself able to forgive. Here it might appear that the victim initially forgives the counsellor (as a stand-in for the offender) and hence that forgiveness travels through a proxy who is *known* by the victim to be a proxy. But that, I suggest, is not actually what happens. For in so far as the victim recognises that the counsellor is not the real offender, she cannot *forgive* the counsellor for a deed which she knows he did not do. Instead, the counsellor’s performance shows the offender to the victim in a new light, delivering an imaginative jolt which frees her from the clutches of her resentment and makes forgiveness possible. While the counsellor plays a vital intermediary role in the process, it is that of a facilitator of forgiveness rather than a proxy through whom forgiveness passes from victim to offender. Moved by the counsellor’s simulation of him, the victim now forgives the offender directly.

## Footnotes

1 I am grateful to Antony Bash, Paula Satne and an anonymous reviewer for the journal for invaluable comments on earlier drafts of this essay.
